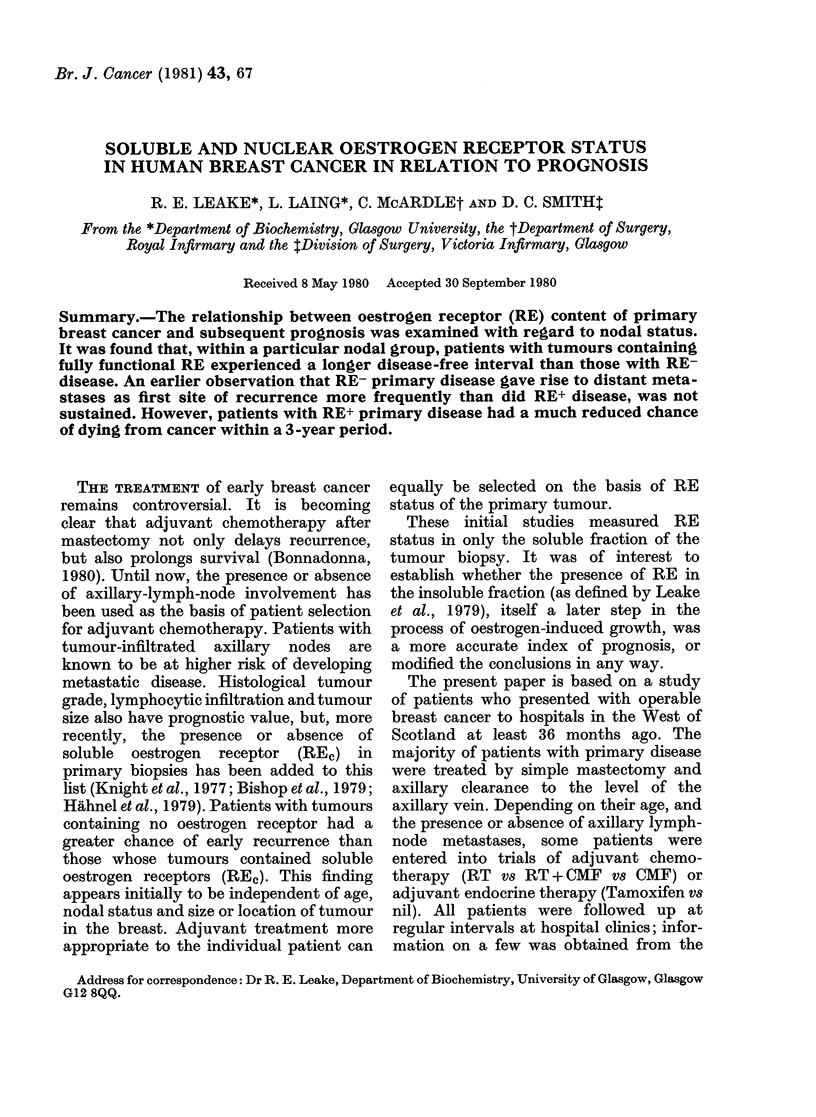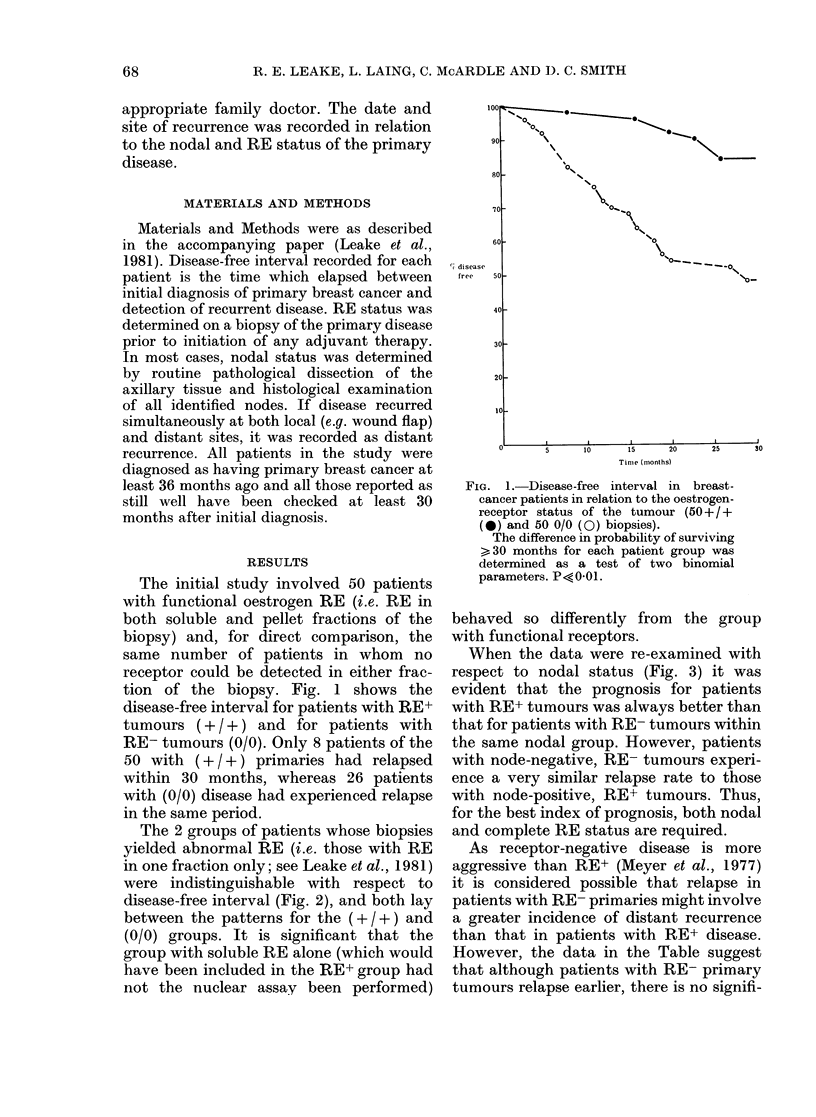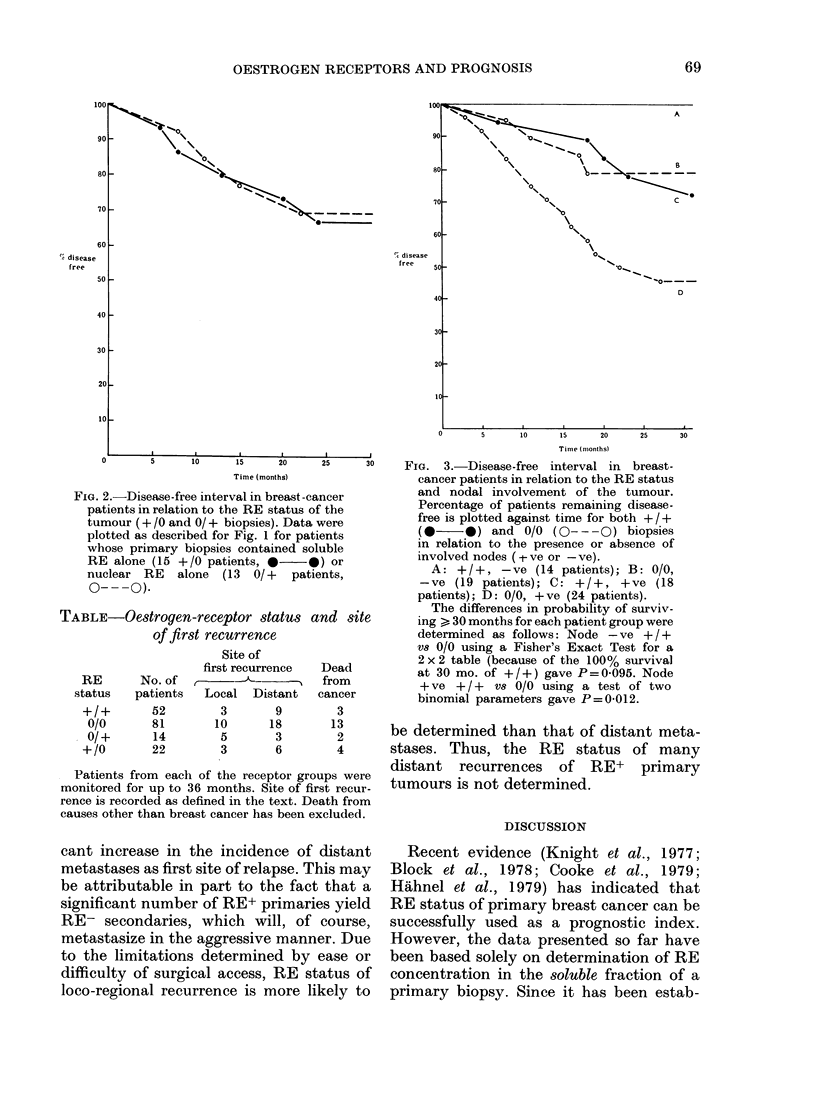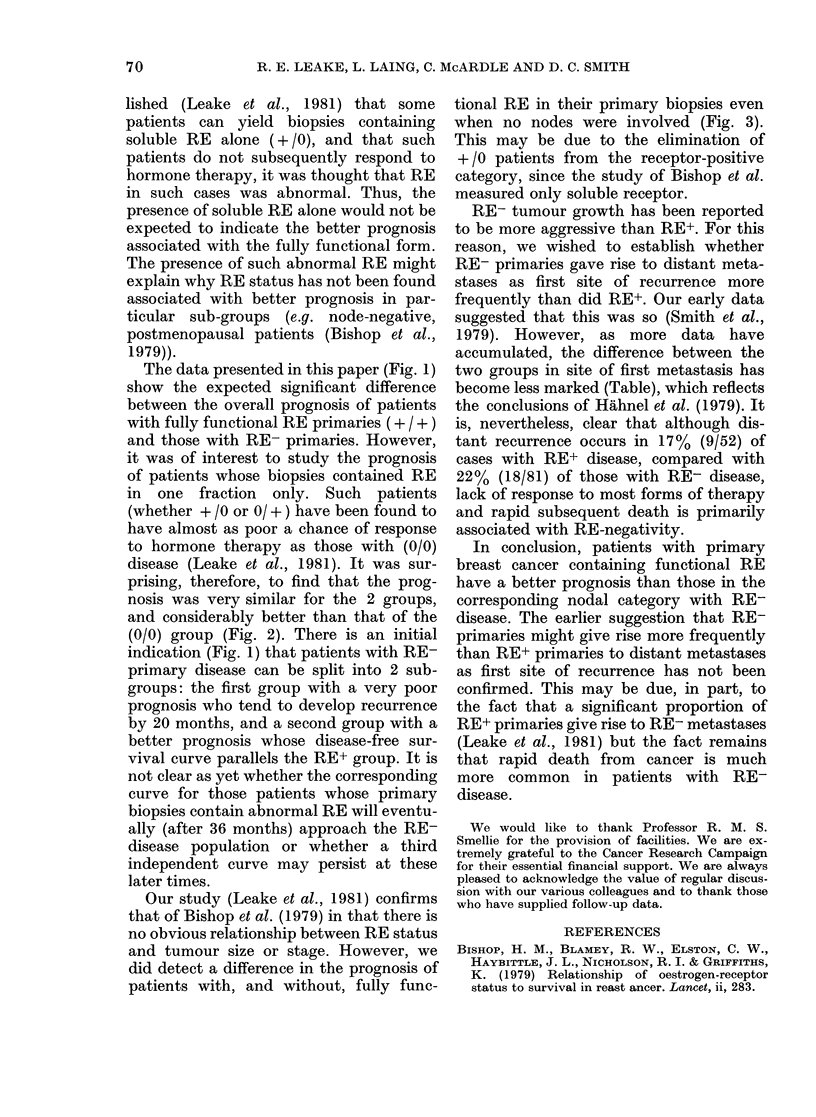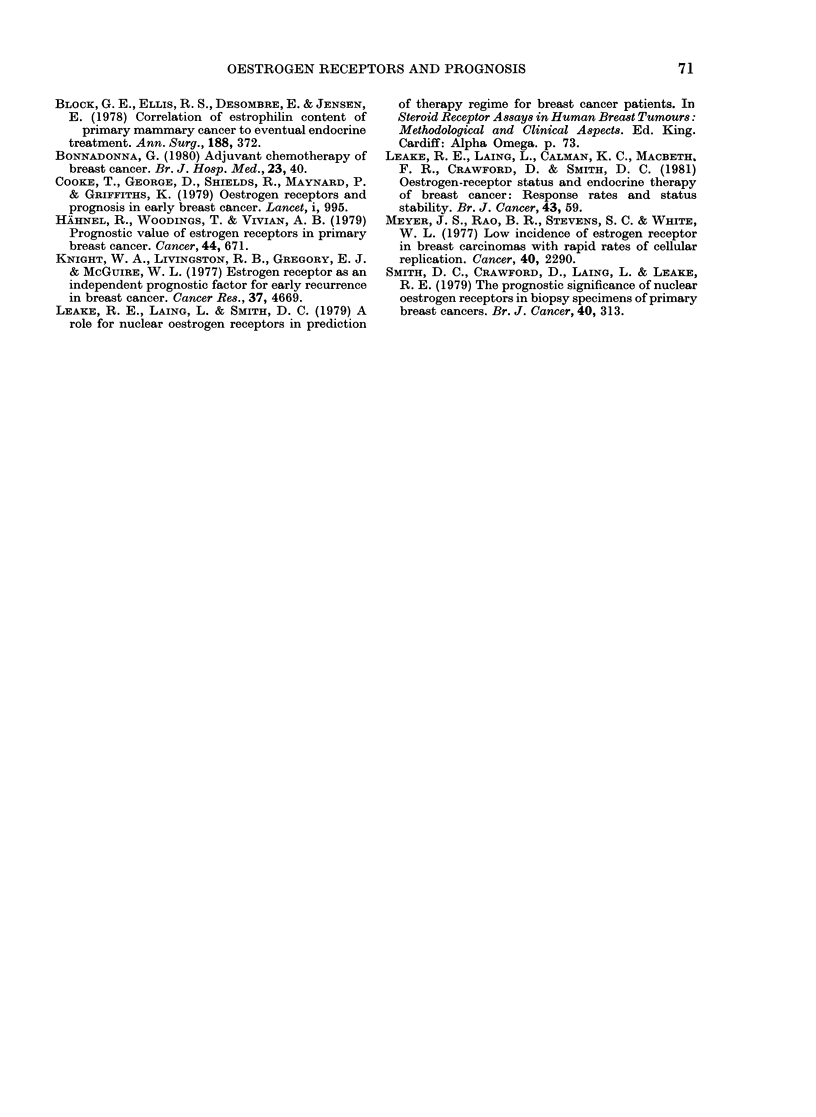# Soluble and nuclear oestrogen receptor status in human breast cancer in relation to prognosis.

**DOI:** 10.1038/bjc.1981.9

**Published:** 1981-01

**Authors:** R. E. Leake, L. Laing, C. McArdle, D. C. Smith

## Abstract

The relationship between oestrogen receptor (RE) content of primary breast cancer and subsequent prognosis was examined with regard to nodal status. It was found that, within a particular nodal group, patients with tumours containing fully functional RE experienced a longer disease-free interval than those with RE- disease. An earlier observation that RE- primary disease gave rise to distant metastases as first site of recurrence more frequently than did RE+ disease, was not sustained. However, patients with RE+ primary disease had a much reduced chance of dying from cancer within a 3-year period.


*Br. J. Cancer* (1981) **43**, 67

### SOLUBLE AND NUCLEAR OESTROGEN RECEPTOR STATUS IN HUMAN BREAST CANCER IN RELATION TO PROGNOSIS

R. E. LEAKE*, L. LAING*, C. McARDLE† AND D. C. SMITH‡

*From the *Department of Biochemistry, Glasgow University, the †Department of Surgery, Royal Infirmary and the ‡Division of Surgery, Victoria Infirmary, Glasgow*

Received 8 May 1980 Accepted 30 September 1980

**Summary.—The relationship between oestrogen receptor (RE) content of primary breast cancer and subsequent prognosis was examined with regard to nodal status. It was found that, within a particular nodal group, patients with tumours containing fully functional RE experienced a longer disease-free interval than those with $RE^-$ disease. An earlier observation that $RE^-$ primary disease gave rise to distant metastases as first site of recurrence more frequently than did $RE^+$ disease, was not sustained. However, patients with $RE^+$ primary disease had a much reduced chance of dying from cancer within a 3-year period.**

THE TREATMENT of early breast cancer remains controversial. It is becoming clear that adjuvant chemotherapy after mastectomy not only delays recurrence, but also prolongs survival (Bonnadonna, 1980). Until now, the presence or absence of axillary-lymph-node involvement has been used as the basis of patient selection for adjuvant chemotherapy. Patients with tumour-infiltrated axillary nodes are known to be at higher risk of developing metastatic disease. Histological tumour grade, lymphocytic infiltration and tumour size also have prognostic value, but, more recently, the presence or absence of soluble oestrogen receptor ($RE_c$) in primary biopsies has been added to this list (Knight *et al.*, 1977; Bishop *et al.*, 1979; Hähnel *et al.*, 1979). Patients with tumours containing no oestrogen receptor had a greater chance of early recurrence than those whose tumours contained soluble oestrogen receptors ($RE_c$). This finding appears initially to be independent of age, nodal status and size or location of tumour in the breast. Adjuvant treatment more appropriate to the individual patient can equally be selected on the basis of RE status of the primary tumour.

These initial studies measured RE status in only the soluble fraction of the tumour biopsy. It was of interest to establish whether the presence of RE in the insoluble fraction (as defined by Leake *et al.*, 1979), itself a later step in the process of oestrogen-induced growth, was a more accurate index of prognosis, or modified the conclusions in any way.

The present paper is based on a study of patients who presented with operable breast cancer to hospitals in the West of Scotland at least 36 months ago. The majority of patients with primary disease were treated by simple mastectomy and axillary clearance to the level of the axillary vein. Depending on their age, and the presence or absence of axillary lymph-node metastases, some patients were entered into trials of adjuvant chemotherapy (RT *vs* RT+CMF *vs* CMF) or adjuvant endocrine therapy (Tamoxifen *vs* nil). All patients were followed up at regular intervals at hospital clinics; information on a few was obtained from the

Address for correspondence: Dr R. E. Leake, Department of Biochemistry, University of Glasgow, Glasgow G12 8QQ.

appropriate family doctor. The date and site of recurrence was recorded in relation to the nodal and RE status of the primary disease.

### MATERIALS AND METHODS

Materials and Methods were as described in the accompanying paper (Leake *et al.*, 1981). Disease-free interval recorded for each patient is the time which elapsed between initial diagnosis of primary breast cancer and detection of recurrent disease. RE status was determined on a biopsy of the primary disease prior to initiation of any adjuvant therapy. In most cases, nodal status was determined by routine pathological dissection of the axillary tissue and histological examination of all identified nodes. If disease recurred simultaneously at both local (*e.g.* wound flap) and distant sites, it was recorded as distant recurrence. All patients in the study were diagnosed as having primary breast cancer at least 36 months ago and all those reported as still well have been checked at least 30 months after initial diagnosis.

### RESULTS

The initial study involved 50 patients with functional oestrogen RE (*i.e.* RE in both soluble and pellet fractions of the biopsy) and, for direct comparison, the same number of patients in whom no receptor could be detected in either fraction of the biopsy. Fig. 1 shows the disease-free interval for patients with $RE^+$ tumours (+/+) and for patients with $RE^-$ tumours (0/0). Only 8 patients of the 50 with (+/+) primaries had relapsed within 30 months, whereas 26 patients with (0/0) disease had experienced relapse in the same period.

The 2 groups of patients whose biopsies yielded abnormal RE (*i.e.* those with RE in one fraction only; see Leake *et al.*, 1981) were indistinguishable with respect to disease-free interval (Fig. 2), and both lay between the patterns for the (+/+) and (0/0) groups. It is significant that the group with soluble RE alone (which would have been included in the $RE^+$ group had not the nuclear assay been performed)

FIG. 1.—Disease-free interval in breast-cancer patients in relation to the oestrogen-receptor status of the tumour (50+/+ (●) and 50 0/0 (○) biopsies).
The difference in probability of surviving ⩾30 months for each patient group was determined as a test of two binomial parameters. $P \ll 0{\cdot}01$.

behaved so differently from the group with functional receptors.

When the data were re-examined with respect to nodal status (Fig. 3) it was evident that the prognosis for patients with $RE^+$ tumours was always better than that for patients with $RE^-$ tumours within the same nodal group. However, patients with node-negative, $RE^-$ tumours experience a very similar relapse rate to those with node-positive, $RE^+$ tumours. Thus, for the best index of prognosis, both nodal and complete RE status are required.

As receptor-negative disease is more aggressive than $RE^+$ (Meyer *et al.*, 1977) it is considered possible that relapse in patients with $RE^-$ primaries might involve a greater incidence of distant recurrence than that in patients with $RE^+$ disease. However, the data in the Table suggest that although patients with $RE^-$ primary tumours relapse earlier, there is no signifi-

FIG. 2.—Disease-free interval in breast-cancer patients in relation to the RE status of the tumour (+/0 and 0/+ biopsies). Data were plotted as described for Fig. 1 for patients whose primary biopsies contained soluble RE alone (15 +/0 patients, ●——●) or nuclear RE alone (13 0/+ patients, ○- - -○).

TABLE—*Oestrogen-receptor status and site of first recurrence*

| RE status | No. of patients | Site of first recurrence: Local | Site of first recurrence: Distant | Dead from cancer |
|---|---|---|---|---|
| +/+ | 52 | 3 | 9 | 3 |
| 0/0 | 81 | 10 | 18 | 13 |
| 0/+ | 14 | 5 | 3 | 2 |
| +/0 | 22 | 3 | 6 | 4 |

Patients from each of the receptor groups were monitored for up to 36 months. Site of first recurrence is recorded as defined in the text. Death from causes other than breast cancer has been excluded.

cant increase in the incidence of distant metastases as first site of relapse. This may be attributable in part to the fact that a significant number of RE⁺ primaries yield RE⁻ secondaries, which will, of course, metastasize in the aggressive manner. Due to the limitations determined by ease or difficulty of surgical access, RE status of loco-regional recurrence is more likely to

FIG. 3.—Disease-free interval in breast-cancer patients in relation to the RE status and nodal involvement of the tumour. Percentage of patients remaining disease-free is plotted against time for both +/+ (●——●) and 0/0 (○- - -○) biopsies in relation to the presence or absence of involved nodes (+ve or −ve).

A: +/+, −ve (14 patients); B: 0/0, −ve (19 patients); C: +/+, +ve (18 patients); D: 0/0, +ve (24 patients).

The differences in probability of surviving ≥30 months for each patient group were determined as follows: Node −ve +/+ *vs* 0/0 using a Fisher's Exact Test for a 2×2 table (because of the 100% survival at 30 mo. of +/+) gave $P=0.095$. Node +ve +/+ *vs* 0/0 using a test of two binomial parameters gave $P=0.012$.

be determined than that of distant metastases. Thus, the RE status of many distant recurrences of RE⁺ primary tumours is not determined.

### DISCUSSION

Recent evidence (Knight *et al.*, 1977; Block *et al.*, 1978; Cooke *et al.*, 1979; Hähnel *et al.*, 1979) has indicated that RE status of primary breast cancer can be successfully used as a prognostic index. However, the data presented so far have been based solely on determination of RE concentration in the *soluble* fraction of a primary biopsy. Since it has been established (Leake *et al.*, 1981) that some patients can yield biopsies containing soluble RE alone (+/0), and that such patients do not subsequently respond to hormone therapy, it was thought that RE in such cases was abnormal. Thus, the presence of soluble RE alone would not be expected to indicate the better prognosis associated with the fully functional form. The presence of such abnormal RE might explain why RE status has not been found associated with better prognosis in particular sub-groups (*e.g.* node-negative, postmenopausal patients (Bishop *et al.*, 1979)).

The data presented in this paper (Fig. 1) show the expected significant difference between the overall prognosis of patients with fully functional RE primaries (+/+) and those with $RE^-$ primaries. However, it was of interest to study the prognosis of patients whose biopsies contained RE in one fraction only. Such patients (whether +/0 or 0/+) have been found to have almost as poor a chance of response to hormone therapy as those with (0/0) disease (Leake *et al.*, 1981). It was surprising, therefore, to find that the prognosis was very similar for the 2 groups, and considerably better than that of the (0/0) group (Fig. 2). There is an initial indication (Fig. 1) that patients with $RE^-$ primary disease can be split into 2 subgroups: the first group with a very poor prognosis who tend to develop recurrence by 20 months, and a second group with a better prognosis whose disease-free survival curve parallels the $RE^+$ group. It is not clear as yet whether the corresponding curve for those patients whose primary biopsies contain abnormal RE will eventually (after 36 months) approach the $RE^-$ disease population or whether a third independent curve may persist at these later times.

Our study (Leake *et al.*, 1981) confirms that of Bishop *et al.* (1979) in that there is no obvious relationship between RE status and tumour size or stage. However, we did detect a difference in the prognosis of patients with, and without, fully functional RE in their primary biopsies even when no nodes were involved (Fig. 3). This may be due to the elimination of +/0 patients from the receptor-positive category, since the study of Bishop *et al.* measured only soluble receptor.

$RE^-$ tumour growth has been reported to be more aggressive than $RE^+$. For this reason, we wished to establish whether $RE^-$ primaries gave rise to distant metastases as first site of recurrence more frequently than did $RE^+$. Our early data suggested that this was so (Smith *et al.*, 1979). However, as more data have accumulated, the difference between the two groups in site of first metastasis has become less marked (Table), which reflects the conclusions of Hähnel *et al.* (1979). It is, nevertheless, clear that although distant recurrence occurs in 17% (9/52) of cases with $RE^+$ disease, compared with 22% (18/81) of those with $RE^-$ disease, lack of response to most forms of therapy and rapid subsequent death is primarily associated with RE-negativity.

In conclusion, patients with primary breast cancer containing functional RE have a better prognosis than those in the corresponding nodal category with $RE^-$ disease. The earlier suggestion that $RE^-$ primaries might give rise more frequently than $RE^+$ primaries to distant metastases as first site of recurrence has not been confirmed. This may be due, in part, to the fact that a significant proportion of $RE^+$ primaries give rise to $RE^-$ metastases (Leake *et al.*, 1981) but the fact remains that rapid death from cancer is much more common in patients with $RE^-$ disease.

We would like to thank Professor R. M. S. Smellie for the provision of facilities. We are extremely grateful to the Cancer Research Campaign for their essential financial support. We are always pleased to acknowledge the value of regular discussion with our various colleagues and to thank those who have supplied follow-up data.

### REFERENCES

BISHOP, H. M., BLAMEY, R. W., ELSTON, C. W., HAYBITTLE, J. L., NICHOLSON, R. I. & GRIFFITHS, K. (1979) Relationship of oestrogen-receptor status to survival in reast ancer. *Lancet*, ii, 283.

BLOCK, G. E., ELLIS, R. S., DESOMBRE, E. & JENSEN, E. (1978) Correlation of estrophilin content of primary mammary cancer to eventual endocrine treatment. *Ann. Surg.*, **188**, 372.

BONNADONNA, G. (1980) Adjuvant chemotherapy of breast cancer. *Br. J. Hosp. Med.*, **23**, 40.

COOKE, T., GEORGE, D., SHIELDS, R., MAYNARD, P. & GRIFFITHS, K. (1979) Oestrogen receptors and prognosis in early breast cancer. *Lancet*, i, 995.

HÄHNEL, R., WOODINGS, T. & VIVIAN, A. B. (1979) Prognostic value of estrogen receptors in primary breast cancer. *Cancer*, **44**, 671.

KNIGHT, W. A., LIVINGSTON, R. B., GREGORY, E. J. & MCGUIRE, W. L. (1977) Estrogen receptor as an independent prognostic factor for early recurrence in breast cancer. *Cancer Res.*, **37**, 4669.

LEAKE, R. E., LAING, L. & SMITH, D. C. (1979) A role for nuclear oestrogen receptors in prediction of therapy regime for breast cancer patients. In *Steroid Receptor Assays in Human Breast Tumours: Methodological and Clinical Aspects*. Ed. King. Cardiff: Alpha Omega. p. 73.

LEAKE, R. E., LAING, L., CALMAN, K. C., MACBETH, F. R., CRAWFORD, D. & SMITH, D. C. (1981) Oestrogen-receptor status and endocrine therapy of breast cancer: Response rates and status stability. *Br. J. Cancer*, **43**, 59.

MEYER, J. S., RAO, B. R., STEVENS, S. C. & WHITE, W. L. (1977) Low incidence of estrogen receptor in breast carcinomas with rapid rates of cellular replication. *Cancer*, **40**, 2290.

SMITH, D. C., CRAWFORD, D., LAING, L. & LEAKE, R. E. (1979) The prognostic significance of nuclear oestrogen receptors in biopsy specimens of primary breast cancers. *Br. J. Cancer*, **40**, 313.